# Finding sexual partners online: prevalence and associations with sexual behaviour, STI diagnoses and other sexual health outcomes in the British population

**DOI:** 10.1136/sextrans-2016-052994

**Published:** 2017-04-10

**Authors:** Melissa Cabecinha, Catherine H Mercer, Kirsten Gravningen, Catherine Aicken, Kyle G Jones, Clare Tanton, Kaye Wellings, Pam Sonnenberg, Nigel Field

**Affiliations:** 1 Research Department of Infection & Population Health, University College London, London, UK; 2 Department of Microbiology and Infection Control, University Hospital of Northern Norway, Norway; 3 Department of Social & Environmental Health Research, London School of Hygiene and Tropical Medicine, London, UK

**Keywords:** SEXUAL BEHAVIOUR, SEXUAL EXPERIENCE, SEXUAL HEALTH

## Abstract

**Objectives:**

Online venues might facilitate sexual encounters, but the extent to which finding partners online is associated with sexual risk behaviour and sexual health outcomes is unclear. We describe use of the internet to find sexual partners in a representative sample in Britain.

**Methods:**

The third National Survey of Sexual Attitudes and Lifestyles (Natsal-3) was a cross-sectional probability survey of 15 162 adults (aged 16–74 years) undertaken 2010–2012. We estimated prevalence of, and identified factors associated with, finding sexual partners online among those reporting ≥1 new sexual partners in the past year.

**Results:**

Finding sexual partners online in the past year was reported by 17.6% (95% CI 15.6 to 19.9) of men and 10.1% (8.5–11.9) of women, and most common among those aged 35–44 years. After age-adjustment, those reporting a non-heterosexual identity were more likely to report this. Finding partners online was also associated with reporting sexual risk behaviours: condomless sex with ≥2 partners (adjusted OR (aOR) men: 1.52 (1.03 to 2.23); women: 1.62 (1.06 to 2.49)), concurrent partnerships (aOR men: 2.33 (1.62 to 3.35); women: 2.41 (1.49 to 3.87)) and higher partner numbers (reporting ≥5 partners aOR men: 5.95 (3.78 to 9.36); women: 7.00 (3.77 to 13.00)) (all past year). STI diagnoses and HIV testing were more common among men reporting finding partners online (adjusted for age, partner numbers, same-sex partnerships), but not women.

**Conclusions:**

Finding partners online was associated with markers of sexual risk, which might be important for clinical risk assessment, but this was not matched by uptake of sexual health services. Online opportunities to find partners have increased, so these data might underestimate the importance of this social phenomenon for public health and STI control.

## Introduction

Sexual partner numbers and sexual mixing patterns are important epidemiological drivers of transmission and persistence of STIs in populations.^1^ ^2^ By facilitating sexual encounters, online venues for meeting partners provide opportunities to increase the rate of partner acquisition, and potentially enable individuals in different social and sexual networks, with varying risk behaviour and STI prevalence, to meet in ways that would not otherwise have happened.^3^ In 2000, an estimated 25% of households in the Great Britain had internet access, and this number steadily increased to 73% of households in 2010, 77% in 2011 and 80% in 2012. In 2016, an estimated 89% of households had internet access, with 75% of individuals having accessed the internet on a mobile device in the last 3 months.^4^ Alongside this rise in internet access, the number of online venues for meeting partners has also increased, but whether using these venues lead to an increased likelihood of poor sexual health outcomes is poorly understood.

In San Francisco, a 1999 syphilis outbreak among men who have sex with men (MSM) was traced back to an online chatroom.^5^ This prompted the internet to be deemed a ‘risk environment’ for STIs;^6^ however, subsequent studies have provided mixed evidence as to whether using the internet to meet sexual partners is a risk environment, and if so whether it is inherently risky, or if seeking sex online is instead a marker for sexual risk behaviour in general.^3^ ^7^ ^w1 w2^

Use of the internet to find sexual partners has frequently been described in MSM populations,^8^ ^w3–w5^ while less is known about finding partners online among heterosexuals. Studies investigating this issue have predominately been conducted in unrepresentative samples, with research mainly carried out in clinic studies,^7^ ^9^ ^w6^ online convenience surveys,^10^ or among youth.^11^ ^w5, w7^ The need for studies in the general adult population has been identified^11^ ^12^ and, to our knowledge, there are no population-level data on internet-use to find sexual partners and its associations with sexual behaviour and STI.

In this study, data from the third National Survey of Sexual Attitudes and Lifestyle (Natsal-3), a national probability sample survey conducted 2010–2012, were used to: (a) estimate the prevalence of using the internet to find sexual partners among men and women in Britain and (b) examine associations between reporting use of the internet to find partners and sociodemographic factors, sexual behaviour and sexual health outcomes.

## Methods

### Study design

Natsal-3 is a multistage, clustered and stratified probability survey of 15 162 men and women aged 16–74 years in Britain.^13^ Participants were interviewed in their homes between August 2010 and August 2012. The response rate was 57.7% and the cooperation rate (of eligible addresses contacted) was 65.8%. Interviews were carried out via computer-assisted personal interviews, with participants completing computer-assisted self-interviews (CASI) to answer more sensitive questions. Full details of the methodology have been published elsewhere.^14^ ^15^

Participants reporting at least one sexual partner over their lifetime were asked the question “Have you used the internet to find a sexual partner in the past twelve months?” in the CASI. The non-response rate for this question was 2.4%. The Natsal-3 questionnaire also asked about a broad range of sociodemographic factors and sexual and health behaviours.

After the interview, we invited a sample of participants aged 16–44 years to provide urine for STI testing. Full methodological details have been described elsewhere.^14^ ^16^ ^w8–w10^

### Statistical analysis

Analysis was performed using Stata (V.14.1), accounting for stratification and clustering.^14^ ^15^ The sample was weighted to account for selection probability and non-response, and corrected for differences in gender, age and regional distribution according to the UK 2011 census, so that the data are broadly representative of the general British population.^15^

The prevalence of using the internet to find sexual partners in the past year, and associated sociodemographic characteristics, were estimated for men and women reporting one or more new sexual partners in the past year (1702 men (weighted population prevalence: 20.7%) and 1776 women (weighted population prevalence: 15.0%)). This group was selected in order to focus on the population for whom this would be most relevant by excluding those in stable relationships and/or those not seeking a partner. We also present estimates for the wider sexually experienced population, defined as participants reporting at least one sexual partner, ever (5698 men (weighted population prevalence: 95.6%) and 7160 women (weighted population prevalence: 96.1%)), to consider the population as a whole.

Use of the internet to find sexual partners was initially treated as the dependent (outcome) variable. Binary logistic regression was used to identify associations between using the internet to find sexual partners and sociodemographic factors, sexual risk behaviours and HIV/STI risk perception. Then, internet-use to find partners was treated as the independent (exposure) variable when examining how reporting of key sexual health outcomes varied according to whether or not participants used the internet to find partners. Crude ORs and 95% CIs were presented. Multivariable logistic regression was used to present adjusted OR (aOR) for key factors associated with sexual health outcomes and reporting using the internet to find partners in the univariable analysis.

## Results

### Comparison of characteristics of men and women reporting using the internet to find sexual partners in the past year

Among participants reporting at least one new sexual partner in the past year, approximately 8% more men than women reported using the internet to find sexual partners in the past year (weighted prevalence 17.6% (15.6 to 19.9) of men (table 1) and 10.1% (8.5 to 11.9) of women (table 2); (age-adjusted OR women: 0.54 (0.4 to 0.7). Participants aged 35–44 years were most likely to report doing so (30.6% (22.7 to 39.7) of men and 17.7% (12.2 to 24.9) of women), while reporting internet-use to find partners was lowest in the youngest age group (10.1% (8.1 to 12.6) of men and 4.2% (3.0 to 5.8) of women aged 16–24 years).

**Table 1:** Variations in reporting finding sexual partners on the internet by key sociodemographic and health behaviours in men reporting one or more new sexual partners in the past year and those reporting at least one sexual partner, ever (2010–2012)

	Men reporting one or more new sexual partner, past year (n=1702)				Men reporting one or more sexual partner, ever (n=5698)			
	Prevalence	Crude OR	Adjusted OR*		Prevalence	Crude OR	Adjusted OR*	
	% (95% CI†)	(95% CI)	(95% CI)	Denominator‡	% (95% CI†)	(95% CI)	(95% CI)	Denominator‡
Overall	17.6 (15.6 to 19.9)			1702, 1480	5.2 (4.7 to 5.8)			5698, 6956
Sociodemographic characteristics								
Age group (years)		p<0.0001	–			p<0.0001	–	
16–24	10.1 (8.1 to 12.6)	1.00	–	818, 573	7.9 (6.5 to 9.7)	1.00	–	1375, 1003
25–34	21.22 (17.0 to 26.1)	2.38 (1.64 to 3.45)	–	473, 372	7.6 (6.2 to 9.3)	0.95 (0.69 to 1.30)	–	1455, 1302
35–44	30.6 (22.7 to 39.7)	3.9 (2.42 to 6.30)	–	145, 194	5.7 (4.3 to 7.5)	0.7 (0.48 to 1.01)	–	788, 1392
45–54	22.2 (15.0 to 31.7)	2.54 (1.47 to 4.38)	–	128, 175	4.0 (2.8 to 5.6)	0.48 (0.31 to 0.73)	–	760, 1359
55–64	16.0 (9.6 to 25.4)	1.69 (0.90 to 3.17)	–	103, 123	3.4 (2.3 to 4.9)	0.41 (0.26 to 0.63)	–	713, 1116
65–74	14.8 (6.2 to 31.3)	1.54 (0.58 to 4.08)	–	35, 43	1.6 (0.8 to 3.1)	0.19 (0.09 to 0.39)	–	607, 785
Ethnicity		p=0.9254	p=0.9627			p=0.0148	p=0.1196	
White	17.8 (15.6 to 20.2)	1.00	1.00	1482, 1250	4.8 (4.3 to 5.5)	1.00	1.00	5110, 6154
Asian	17.8 (8.2 to 34.2)	1 (0.41 to 2.42)	1.05 (0.44 to 2.51)	70, 75	6.7 (4.0 to 11.1)	1.41 (0.81 to 2.48)	1.27 (0.72 to 2.25)	247, 389
Black	17.1 (9.6 to 28.7)	0.96 (0.49 to 1.88)	0.9 (0.45 to 1.80)	78, 87	9.2 (5.6 to 14.8)	1.99 (1.15 to 3.45)	1.79 (1.03 to 3.10)	162, 212
Mixed/other	14.1 (6.8 to 26.9)	0.76 (0.34 to 1.70)	0.85 (0.38 to 1.90)	70, 65	8.77 (4.9 to 14.9)	1.87 (0.99 to 3.51)	1.47 (0.77 to 2.81)	165, 181
NSSEC code (individual socioeconomic status)§		p<0.0001	p=0.0001			p=0.173	p=0.6051	
Managerial and professional occupations	26.6 (21.5 to 32.3)	1.00	1.00	383, 389	5.3 (4.3 to 6.6)	1.00	1.00	1817, 2552
Intermediate occupations	21.1 (15.6 to 28.0)	0.74 (0.46 to 1.19)	0.75 (0.46 to 1.20)	222, 216	4.7 (3.5 to 6.2)	0.87 (0.60 to 1.27)	0.88 (0.61 to 1.29)	891, 1183
Semi-routine/routine occupations	14.8 (11.9 to 18.2)	0.48 (0.33 to 0.70)	0.5 (0.34 to 0.73)	637, 524	5.3 (4.4 to 6.4)	0.99 (0.73 to 1.33)	0.88 (0.65 to 1.19)	1974, 2281
No job (10+ hours/week) or not in last 10 years	9.9 (5.1 to 18.3)	0.30 (0.14 to 0.65)	0.30 (0.14 to 0.65)	81, 62	3.4 (2.1 to 5.5)	0.63 (0.36 to 1.08)	0.81 (0.46 to 1.40)	398, 436
Student in full-time education	9.4 (6.5 to 13.3)	0.29 (0.18 to 0.46)	0.33 (0.19 to 0.57)	374, 284	7.0 (5.0 to 9.7)	1.33 (0.87 to 2.03)	0.69 (0.43 to 1.10)	595, 476
Quintiles of IMD¶		p=0.7208	p=0.7316			p=0.0775	p=0.2789	
1 (least deprived)	18.2 (13.4 to 24.2)	1.00	1.00	294, 259	4.3 (3.2 to 5.8)	1.00	1.00	1118, 1443
2	21.0 (15.3 to 28.1)	1.2 (0.69 to 2.06)	1.29 (0.75 to 2.25)	289, 252	5.0 (3.8 to 6.7)	1.16 (0.76 to 1.79)	1.14 (0.74 to 1.75)	1134, 1484
3	15.6 (11.6 to 20.7)	0.83 (0.51 to 1.35)	0.9 (0.54 to 1.48)	320, 271	4.88 (3.8 to 6.2)	1.12 (0.75 to 1.67)	1.05 (0.70 to 1.56)	1110, 1358
4	17.0 (13.2 to 21.7)	0.92 (0.58 to 1.46)	0.99 (0.62 to 1.58)	358, 329	5.0 (4.0 to 6.4)	1.17 (0.79 to 1.73)	1.03 (0.70 to 1.53)	1133, 1372
5 (most deprived)	17.0 (13.2 to 21.6)	0.92 (0.57 to 1.47)	0.97 (0.60 to 1.57)	441, 368	6.9 (5.6 to 8.5)	1.62 (1.11 to 2.37)	1.41 (0.96 to 2.06)	1203, 1300
Population density of residence**		p=0.1113	p=0.1441			p=0.1852	p=0.7341	
Rural or town area	21.1 (16.6 to 26.5)	1.00	1.00	314, 259	4.5 (3.5 to 5.7)	1.00	1.00	1257, 1610
Urban area	16.9 (14.6 to 19.4)	0.76 (0.54 to 1.07)	0.78 (0.55 to 1.09)	1388, 1221	5.4 (4.8 to 6.1)	1.21 (0.91 to 1.60)	1.05 (0.79 to 1.40)	4441, 5346
Internet access at home		p=0006	p=0.0001			p=0.2892	p=0.9365	
No	7.5 (4.4 to 12.6)	1.00	1.00	186, 148	4.3 (2.9 to 6.2)	1.00	1.00	683, 690
Yes	18.7 (16.5 to 21.2)	2.84 (1.57 to 5.14)	3.43 (1.85 to 6.36)	1514, 1330	5.3 (4.7 to 6.0)	1.26 (0.82 to 1.93)	1.02 (0.66 to 1.57)	5005, 6249
Sexual identity		p<0.0001	p<0.0001			p<0.0001	p<0.0001	
Heterosexual/straight	15.1 (13.1 to 17.3)	1.00	1.00	1610, 1398	4.5 (4.0 to 5.1)	1.00	1.00	5494, 6747
Gay/lesbian	68.8 (53.6 to 80.8)	12.41 (6.45 to 23.86)	12.75 (6.53 to 24.88)	57, 49	36.2 (26.4 to 47.4)	12.1 (7.55 to 19.41)	11.1 (6.96 to 17.72)	116, 108
Bisexual	45.90 (26.4 to 66.7)	4.76 (2.05 to 11.05)	4.38 (1.88 to 10.22)	30, 29	22.1 (12.7 to 35.7)	6.04 (3.08 to 11.83)	5.99 (2.96 to 12.12)	63, 73
Relationship status at time of interview		p=0.1199	p=0.0542			p<0.0001	p<0.0001	
Married/civil partnership	18.2 (11.8 to 27.0)	1.00	1.00	128, 217	1.7 (1.2 to 2.4)	1.00	1.00	2099, 3661
Living with partner	14.2 (8.4 to 22.9)	0.74 (0.34 to 1.65)	0.93 (0.41 to 2.12)	145, 153	3.7 (2.5 to 5.4)	2.24 (1.30 to 3.87)	2.05 (1.17 to 3.60)	868, 1044
In a ‘steady’ ongoing relationship but not living together	14.1 (10.6 to 18.4)	0.74 (0.40 to 1.35)	1.03 (0.54 to 1.98)	509, 395	8.7 (6.8 to 11.1)	5.65 (3.59 to 8.87)	4.97 (3.06 to 8.06)	959, 767
Not in a ‘steady’ relationship	20.1 (17.3 to 23.3)	1.14 (0.66 to 1.95)	1.58 (0.87 to 2.86)	915, 710	13.4 (11.7 to 15.3)	9.16 (6.17 to 13.58)	8.39 (5.60 to 12.58)	1726, 1441
Health behaviours								
Average alcohol consumption, per week††		p=0.1314	p=0.1914			p=0.0759	p=0.0313	
None	15.3 (10.8 to 21.2)	1.00	1.00	280, 266	4.5 (3.4 to 5.9)	1.00	1.00	1106, 1389
Not more than recommended	17.10 (14.8 to 19.6)	1.14 (0.74 to 1.75)	1.2 (0.78 to 1.84)	1247, 1055	5.1 (4.4 to 5.8)	1.13 (0.82 to 1.55)	1.1 (0.80 to 1.52)	4037, 4911
More than recommended	24.00 (17.0 to 32.6)	1.74 (0.98 to 3.12)	1.71 (0.95 to 3.08)	157, 144	7.2 (5.2 to 9.7)	1.63 (1.05 to 2.54)	1.73 (1.11 to 2.70)	529, 630
Current smoker		p=0.6068	p=0.8571			p=0.0002	p=0.0094	
No	18.10 (15.4 to 21.0)	1.00	1.00	1013, 916	4.5 (3.9 to 5.2)	1.00	1.00	3960, 5077
Yes	17.0 (14.0 to 20.4)	0.93 (0.69 to 1.24)	0.97 (0.73 to 1.31)	689, 564	7.0 (5.9 to 8.3)	1.58 (1.25 to 2.00)	1.38 (1.08 to 1.75)	1738, 1879
Drug use, past year		p=0.0180	p=0.0216			p<0.0001	p=0.0001	
None	17.8 (15.2 to 20.6)	1.00	1.00	1087, 991	4.3 (3.8 to 4.9)	1.00	1.00	4523, 5846
Yes, cannabis only	12.5 (9.1 to 17.0)	0.66 (0.44 to 0.98)	0.8 (0.53 to 1.21)	322, 256	7.70 (5.9 to 10.1)	1.86 (1.34 to 2.57)	1.35 (0.95 to 1.92)	639, 605
Yes, drugs other than cannabis	22.6 (17.0 to 29.4)	1.35 (0.90 to 2.02)	1.58 (1.03 to 2.40)	286, 226	12.4 (9.4 to 16.3)	3.15 (2.22 to 4.46)	2.32 (1.59 to 3.38)	512, 473

*Adjusted for age.

†Confidence interval.

‡Unweighted, weighted.

§National Statistics Socio-economic Classification.^17^

¶IMD is a multidimensional measure of area-level deprivation based on participants’ postcode: IMD scores for England, Scotland and Wales were adjusted before being combined and assigned to quintiles, using a method by Payne and Abel.^18^

**Rural <10 000, urban >10 000.

††Recommended alcohol limits (21 units/week for men and 14 units/week for women) as defined by Royal College of Physicians.^19^

IMD, Index of Multiple Deprivation.

**Table 2:** Variations in reporting finding sexual partners on the internet by key sociodemographic and health behaviours in women reporting one or more new sexual partners in the past year and those reporting at least one sexual partner, ever (2010–2012)

	Women reporting one or more new sexual partner, past year (n=1776)				Women reporting one or more sexual partner, ever (n=8198)			
	Prevalence	Crude OR	Adjusted OR*		Prevalence	Crude OR	Adjusted OR*	
	% (95% CI†)	(95% CI)	(95% CI)	Denominator‡	% (95% CI†)	(95% CI)	(95% CI)	Denominator‡
Overall	10.1 (8.5 to 11.9)			1776, 1094	2.4 (2.1 to 2.8)			8198, 7160
Sociodemographic characteristics								
Age group (years)		p<0.0001	–			0.0006	–	
16–24	4.2 (3.0 to 5.8)	1.00	–	845, 461	3.0 (2.3 to 4.0)	1.00	–	1739, 967
25–34	12.8 (9.6 to 16.8)	3.37 (2.07 to 5.48)	–	539, 271	3.6 (2.8 to 4.6)	1.22 (0.82 to 1.80)	–	2393, 1321
35–44	17.7 (12.2 to 24.9)	4.94 (2.76 to 8.84)	–	177, 163	2.8 (2.0 to 3.9)	0.93 (0.59 to 1.48)	–	1182, 1415
45–54	13.2 (8.4 to 20.0)	3.48 (1.89 to 6.41)	–	135, 129	1.9 (1.3 to 2.8)	0.64 (0.39 to 1.03)	–	1088, 1403
55–64	13.2 (5.8 to 27.1)	3.49 (1.36 to 8.96)	–	55, 52	1.6 (1.0 to 2.5)	0.51 (0.29 to 0.91)	–	984, 1185
65–74	–	–	–	Too small to report	1.0 (0.5 to 2.0)	0.32 (0.15 to 0.69)	–	812, 868
Ethnicity		p=0.5350	p=0.4444			p=0.1910	p=0.3827	
White	10.2 (8.5 to 12.2)	1.00	1.00	1543, 933	2.3 (2.0 to 2.7)	1.00	1.00	7339, 6390
Asian	5.1 (1.2 to 19.1)	0.47 (0.11 to 2.03)	0.44 (0.10 to 1.96)	65, 56	2.2 (1.0 to 4.8)	0.96 (0.42 to 2.17)	0.86 (0.38 to 1.96)	323, 311
Black	7.7 (3.2 to 17.4)	0.73 (0.29 to 1.85)	0.82 (0.32 to 2.08)	63, 45	2.3 (1.2 to 4.4)	0.99 (0.49 to 1.99)	0.91 (0.45 to 1.82)	246, 235
Mixed/other	14.7 (6.8 to 29.0)	1.52 (0.64 to 3.60)	1.69 (0.71 to 4.03)	92, 58	5.0 (2.5 to 9.7)	2.22 (1.08 to 4.58)	1.88 (0.91 to 3.91)	272, 208
NSSEC code (individual socioeconomic status)§		p<0.0001	p=0.0074			p=0.3835	p=0.2588	
Managerial and professional occupations	16.2 (12.3 to 21.1)	1.00	1.00	377, 257	2.8 (2.2 to 3.5)	1.00	1.00	2369, 2257
Intermediate occupations	11.7 (7.6 to 17.6)	0.68 (0.39 to 1.21)	0.71 (0.40 to 1.27)	275, 179	2.3 (1.6 to 3.2)	0.83 (0.55 to 1.24)	0.83 (0.55 to 1.25)	1607, 1444
Semi-routine/routine occupations	8.8 (6.3 to 12.3)	0.5 (0.31 to 0.82)	0.56 (0.33 to 0.93)	555, 319	2.3 (1.7 to 3.0)	0.82 (0.56 to 1.21)	0.78 (0.52 to 1.15)	2315, 1902
No job (10+ hours/week) or not in last 10 years	11.4 (6.6 to 18.9)	0.66 (0.33 to 1.31)	0.69 (0.34 to 1.39)	154, 99	1.7 (1.1 to 2.6)	0.61 (0.37 to 1.00)	0.77 (0.47 to 1.28)	1119, 1064
Student in full-time education	3.5 (2.1 to 5.9)	0.19 (0.10 to 0.36)	0.27 (0.13 to 0.55)	394, 234	2.5 (1.6 to 4.0)	0.92 (0.54 to 1.55)	0.54 (0.30 to 0.95)	745, 454
Quintiles of IMD¶		p=0.1616	p=0.1516			p=0.0725	p=0.1151	
1 (least deprived)	9.3 (6.0 to 14.1)	1.00	1.00	257, 167	1.6 (1.1 to 2.4)	1.00	1.00	1504, 1457
2	14.7 (10.1 to 21.1)	1.69 (0.89 to 3.19)	1.77 (0.92 to 3.41)	324, 206	2.9 (2.1 to 4.0)	1.78 (1.07 to 2.97)	1.74 (1.05 to 2.90)	1603, 1492
3	8.0 (5.1 to 12.2)	0.84 (0.44 to 1.63)	0.83 (0.43 to 1.62)	331, 214	2.0 (1.4 to 2.7)	1.21 (0.73 to 2.01)	1.13 (0.68 to 1.88)	1605, 1398
4	10.4 (7.3 to 14.6)	1.13 (0.62 to 2.07)	1.2 (0.64 to 2.23)	401, 249	3.0 (2.2 to 3.9)	1.84 (1.14 to 2.99)	1.66 (1.03 to 2.68)	1705, 1430
5 (most deprived)	8.3 (6.0 to 11.5)	0.89 (0.49 to 1.60)	0.97 (0.53 to 1.78)	454, 258	2.5 (1.9 to 3.3)	1.54 (0.96 to 2.49)	1.37 (0.86 to 2.20)	1781, 1384
Population density of residence**		p=0.8583	p=0.7970			p=0.0260	p=0.0689	
Rural or town area	10.5 (6.6 to 16.1)	1.00	1.00	294, 179	1.7 (1.2 to 2.4)	1.00	1.00	1732, 1640
Urban area	10.0 (8.3 to 12.0)	0.95 (0.56 to 1.62)	1.07 (0.64 to 1.79)	1473, 915	2.6 (2.2 to 3.0)	1.57 (1.06 to 2.35)	1.44 (0.97 to 2.13)	6466, 5520
Internet access at home		p=0.1778	p=0.0820			p=0.1204	p=0.4433	
No	6.7 (3.5 to 12.5)	1.00	1.00	212, 109	1.6 (1.0 to 2.7)	1.00	1.00	1048, 801
Yes	10.5 (8.8 to 12.4)	1.63 (0.80 to 3.30)	1.84 (0.93 to 3.65)	1552, 985	2.5 (2.1 to 2.9)	1.55 (0.89 to 2.69)	1.25 (0.71 to 2.18)	7136, 6345
Sexual identity		p=0.0003	p=0.0001			p<0.0001	p<0.0001	
Heterosexual/straight	9.3 (7.8 to 11.2)	1.00	1.00	1671, 1035	2.2 (1.9 to 2.6)	1.00	1.00	7918, 6949
Gay/lesbian	–	–	–	Too small to report	6.1 (2.7 to 13.3)	2.88 (1.24 to 6.67)	2.69 (1.16 to 6.24)	92, 75
Bisexual	22.1 (12.6 to 35.9)	2.75 (1.36 to 5.55)	3.44 (1.69 to 6.97)	65, 41	10.8 (6.5 to 17.4)	5.3 (2.99 to 9.42)	4.15 (2.32 to 7.43)	150, 102
Relationship status at time of interview		p=0.1637	p=0.0340			p<0.0001	p<0.0001	
Married/civil partnership	7.0 (3.2 to 14.7)	1.00	1.00	118, 127	0.6 (0.4 to 1.0)	1.00	1.00	3089, 3685
Living with partner	7.33 (3.6 to 14.3)	1.06 (0.35 to 3.20)	1.78 (0.59 to 5.42)	195, 140	1.2 (0.6 to 2.2)	1.95 (0.89 to 4.29)	1.65 (0.75 to 3.60)	1311, 1044
In a ‘steady’ ongoing relationship but not living together	9.1 (6.7 to 12.3)	1.34 (0.55 to 3.24)	2.46 (1.00 to 6.05)	627, 341	4.8 (3.7 to 6.4)	8.4 (4.81 to 14.67)	6.72 (3.81 to 11.87)	1375, 797
Not in a ‘steady’ relationship	12.1 (9.9 to 14.8)	1.84 (0.79 to 4.32)	3.14 (1.34 to 7.36)	820, 483	5.9 (4.9 to 7.0)	10.33 (6.21 to 17.17)	9.65 (5.84 to 15.93)	2371, 1599
Health behaviours								
Average alcohol consumption, per week††		p=0.9375	p=0.7692			p=0.1540	p=0.0296	
None	9.5 (6.5 to 13.8)	1.00	1.00	393, 265	1.9 (1.4 to 2.6)	1.00	1.00	2607, 2296
Not more than recommended	10.2 (8.3 to 12.5)	1.08 (0.68 to 1.71)	1.12 (0.70 to 1.77)	1045, 633	2.4 (2.0 to 2.8)	1.25 (0.87 to 1.80)	1.23 (0.86 to 1.77)	4610, 4040
More than recommended	10.5 (6.8 to 15.9)	1.11 (0.59 to 2.08)	1.26 (0.67 to 2.37)	318, 191	3.8 (2.7 to 5.4)	2.03 (1.25 to 3.29)	1.91 (1.18 to 3.08)	950, 799
Current smoker		p=0.1921	p=0.4816			p=0.0147	p=0.0821	
No	10.9 (8.9 to 13.3)	1.00	1.00	1060, 692	2.2 (1.8 to 2.6)	1.00	1.00	5878, 5426
Yes	8.7 (6.6 to 11.15)	0.78 (0.54 to 1.13)	0.87 (0.60 to 1.28)	707, 402	3.1 (2.4 to 3.9)	1.45 (1.08 to 1.95)	1.31 (0.97 to 1.77)	2320, 1733
Drug use, past year		p=0.0658	p=0.0015			p<0.0001	p<0.0001	
None	9.3 (7.6 to 11.3)	1.00	1.00	1381, 876	2.1 (1.7 to 2.4)	1.00	1.00	7395, 6644
Yes, cannabis only	10.8 (6.5 to 17.4)	1.19 (0.66 to 2.15)	1.64 (0.90 to 3.00)	204, 120	5.3 (3.3 to 8.4)	2.66 (1.57 to 4.49)	2.09 (1.21 to 3.62)	440, 288
Yes, drugs other than cannabis	16.8 (10.6 to 25.6)	1.97 (1.11 to 3.51)	2.8 (1.56 to 5.01)	178, 96	8.9 (5.7 to 13.7)	4.66 (2.79 to 7.77)	3.58 (2.11 to 6.07)	338, 207

*Adjusted for age.

‡Unweighted, weighted.

§National Statistics Socio-economic Classification.^17^

¶IMD is a multidimensional measure of area-level deprivation based on participants’ postcode: IMD scores for England, Scotland and Wales were adjusted before being combined and assigned to quintiles, using a method by Payne and Abel.^18^

**Rural <10 000, urban >10 000.

††Recommended alcohol limits (21 units/week for men and 14 units/week for women) as defined by Royal College of Physicians.^19^

IMD, Index of Multiple Deprivation.

After adjusting for age, identifying as non-heterosexual was associated with reporting finding partners online in the main study population. Female participants with no steady partner were more likely to report using the internet to find sexual partners, compared with participants with a steady partner. In regard to National Statistics Socio-economic Classifications (NSSEC),^17^ men and women in full-time education were less likely to have used the internet to find partners than their counterparts in managerial and professional occupations ((aOR 0.33 (0.19 to 0.57) for men, aOR 0.27 (0.13 to 0.55) for women)). For men only, access to the internet at home was associated with using the internet to find partners (aOR 3.43 (1.85 to 6.36)), as was drug use in the past year. For women, there was an association with using the internet to find partners and having used drugs other than cannabis in the past year (aOR 2.8 (1.56 to 5.01).

In the wider sexually experienced population, a lower proportion of participants reported finding partners online (tables 1 and 2). As in the main study population, in the wider sexually experienced population more men than women reported doing so (5.2% (4.7 to 5.8) and 2.4% (2.1 to 2.8), respectively), however, the relationship with age was different; younger participants were more likely to report using the internet to find partners online (7.0% (6.1 to 7.9) of men and 3.2% (2.5 to 4.0) of women aged 16–44 years vs 3.1% (2.7 to 3.7) of men and 1.6% (1.2 to 2.0) of women aged over 45 years).

Associations with sexual identity, relationship status and substance use were also identified in the sexually experienced population for both genders, but the association with NSSEC was observed for women only. There was no association with having access to the internet at home for either gender.

### Associations between sexual behaviour, risk perception and finding partners online

Reporting using the internet to find sexual partners in the past year was associated with reporting sexual risk behaviours among those reporting new partners in the past year (table 3).

**Table 3:** Reporting finding partners online in the past year in relation to sexual behaviour and risk perception, by sex (2010–2012)

	Men reporting one or more new sexual partner, past year (n=1702)				Women reporting one or more new sexual partner, past year (n=1776)			
	Prevalence	Crude OR	Adjusted OR*		Prevalence	Crude OR	Adjusted OR*	
	% (95% CI†)	(95% CI)	(95% CI)	Denominator‡	% (95% C)	(95% CI)	(95% CI)	Denominator‡
Sexual risk behaviour								
No. of sexual partners, past year		p<0.0001	p<0.0001			p<0.0001	p<0.0001	
1	8.8 (6.6 to 11.6)	1.00	1.00	606, 533	6.5 (4.8 to 8.8)	1.00	1.00	733, 495
2	14.7 (11.2 to 19.1)	1.79 (1.15 to 2.79)	1.92 (1.22 to 3.01)	436, 374	8.9 (5.9 to 13.2)	1.4 (0.81 to 2.42)	1.96 (1.11 to 3.49)	451, 267
3–4	22.1 (17.3 to 27.8)	2.96 (1.91 to 4.57)	3.13 (2.00 to 4.90)	374, 326	13.8 (9.9 to 18.8)	2.29 (1.38 to 3.79)	3.65 (2.09 to 6.37)	368, 203
5+	35.3 (28.8 to 42.3)	5.67 (3.65 to 8.82)	5.95 (3.78 to 9.36)	286, 247	20.6 (14.3 to 28.8)	3.73 (2.13 to 6.52)	7.00 (3.77 to 13.00)	212, 127
Same sex partner, past year		p<0.0001	p<0.0001			p= 0.0001	p<0.0001	
No	14.7 (12.8 to 16.9)	1.00	1.00	1604, 1389	9.1 (7.6 to 10.8)	1.00	1.00	1656, 1025
Yes	62.4 (50.5 to 73.0)	9.62 (5.79 to 15.99)	9.12 (5.45 to 15.27)	98, 91	25.2 (16.0 to 37.3)	3.37 (1.85 to 6.13)	3.7 (2.02 to 6.76)	111, 69
Condomless sex with ≥2 partners, past year		p=0.0051				p=0.0205	p= 0.0270	
No	15.8 (13.7 to 18.2)	1.00	1.00	1394, 1179	9.1 (7.4 to 11.0)	1.00	1.00	1347, 849
Yes	24.2 (18.7 to 30.8)	1.7 (1.17 to 2.47)	1.52 (1.03 to 2.23)	296, 288	14.1 (10.2 to 19.1)	1.65 (1.08 to 2.51)	1.62 (1.06 to 2.49)	407, 236
Condomless sex on first occasion with most recent partner		p=0.5590	p=0.8891			p=0.0481	p=0.1561	
No	15.6 (12.7 to 19.1)	1.00	1.00	721, 595	7.22 (5.3 to 9.8)	1.00	1.00	757, 466
Yes	17.1 (13.7 to 21.1)	1.11 (0.78 to 1.59)	1.03 (0.71 to 1.50)	555, 528	10.7 (8.2 to 14.0)	1.54 (1.00 to 2.36)	1.38 (0.88 to 2.14)	639, 397
Concurrent partners, past year		p<0.0001	p<0.0001			p=0.0077	p=0.0015	
No	14.0 (11.5 to 16.8)	1.00	1.00	964, 828	8.4 (6.8 to 10.4)	1.00	1.00	1055, 680
Yes	27.3 (22.2 to 33.1)	2.31 (1.62 to 3.31)	2.33 (1.62 to 3.35)	395, 365	15.9 (11.5 to 21.5)	2.05 (1.30 to 3.23)	2.41 (1.49 to 3.87)	356, 208
≥2 partners, unknown if overlapping	16.0 (12.0 to 21.0)	1.17 (0.79 to 1.75)	1.2 (0.80 to 1.79)	343, 287	9.8 (6.2 to 15.1)	1.18 (0.68 to 2.03)	1.37 (0.78 to 2.38)	356, 207
Taken drugs to assist sexual performance		p<0.0001	p<0.0001			p= 0.0663	p= 0.0323	
No	14.7 (12.7 to 17.0)	1.00	1.00	1437, 1228	9.9 (8.3 to 11.8)	1.00	1.00	1691, 1054
Yes	32.7 (26.1 to 40.0)	2.81 (1.96 to 4.01)	2.56 (1.77 to 3.70)	252, 241	17.6 (9.5 to 30.2)	1.94 (0.96 to 3.95)	2.21 (1.07 to 4.57)	65,34
Paid money for sex, past year§		p<0.0001	p=0.0440			–	–	
No	16.7 (14.9 to 19.2)	1.00	1.00	1642, 1410	–	–	–	–
Yes	33.1 (20.5 to 48.8)	2.43 (1.24 to 4.78)	2.09 (1.02 to 4.28)	57, 66	–	–	–	–
Risk perception								
HIV/AIDS risk: to self		p<0.0001	p<0.0001			p= 0.0001	p<0.0001	
Not at all at risk	12.8 (10.4 to 15.6)	1.00	1.00	793, 704	7.1 (5.3 to 9.3)	1.00	1.00	999, 630
Not very much	2.1 (17.7 to 24.9)	1.82 (1.32 to 2.49)	1.92 (1.38 to 2.66)¶	752, 654	13.5 (10.6 to 16.9)	2.05 (1.37 to 3.07)	2.39 (1.57 to 3.62)	660, 393
Greatly/quite a lot at risk	27.5 (20.0 to 36.6)	2.59 (1.62 to 4.15)	2.68 (1.64 to 4.38)¶	141, 108	18.8 (10.6 to 31.1)	3.05 (1.52 to 6.12)	3.12 (1.48 to 6.59)	99, 66
Other STI risk: to self		p<0.0001				p=0.0004	p<0.0001	
Not at all at risk	12.2 (9.6 to 15.4)	1.00	1.00	655, 595	7.2 (5.4 to 9.6)	1.00	1.00	879, 563
Not very much	18.9 (15.9 to 22.3)	1.68 (1.19 to 2.36)	1.86 (1.30 to 2.67)¶	819, 700	12.2 (9.5 to 15.5)	1.79 (1.19 to 2.71)	2.18 (1.41 to 3.38)	730, 433
Greatly/quite a lot at risk	29.4 (22.4 to 37.5)	2.99 (1.90 to 4.71)	3.36 (2.08 to 5.45)¶	217, 174	17.5 (12.1 to 24.7)	2.73 (1.61 to 4.62)	3.43 (1.94 to 6.06)	145, 91

*Adjusted for age.

‡Unweighted, weighted.

§Outcome reported for men only.

¶p<0.0001.

Those reporting higher partner numbers, condomless sex with two or more sexual partners and overlapping partnerships, all in the past year, were more likely to report using the internet to find sexual partners, and these associations persisted after age-adjustment. For men, there was a particularly strong association between reporting a same-sex partnership in the past year and finding partners online (aOR 9.12 (5.45 to 15.27)). For women, this association was also significant (aOR 3.7 (2.02 to 6.76)). For men, there was also a positive association with reporting paying for sex in the past year and finding partners online (aOR 2.09 (1.02 to 4.28)). Participants describing their risk perception of HIV and/or other STIs acquisition as ‘greatly/quite a lot’ at risk were more likely to report using the internet to find a sexual partner than those perceiving themselves as ‘not at all’ at risk.

In the wider, sexually experienced population, we observed similar but stronger associations between finding partners online and reporting sexual risk behaviours and HIV/STI risk perception for both men and women (see online supplement 1).[Supplementary-material SM2]

10.1136/sextrans-2016-052994.supp1 supplement 1

10.1136/sextrans-2016-052994.supp2 Supplementary appendix

### Associations between using the internet to find sexual partners and sexual health outcomes and health seeking behaviours

Use of the internet to find sexual partners was associated with a range of poor sexual health outcomes, including STI diagnoses in the past year, and sexual health seeking behaviours for men in the age-adjusted analysis (table 4). Reporting higher partner numbers and younger age are both strong predictors of STI risk,^16^ as is reporting a same-sex partner for men,^20^ and the analyses for sexual health outcomes were therefore adjusted for the confounding effect of partner number for both genders, and reporting a same-sex partner for men, in addition to age. After adjusting for the potential confounders of age, partner number and reporting a same-sex partner in the past year, associations persisted for men who reported having had an HIV test in the past year (aOR 2.24 (1.39 to 3.60)) and those diagnosed with an STI in the past year (aOR 2.36 (1.02 to 5.45)). In contrast, associations with having had a test for HIV in the past year and emergency contraception use in the last year were observed for women in the age-adjusted analysis, and no associations persisted after adjustment.

**Table 4:** Reporting of sexual health outcomes and health seeking behaviours in relation to finding partners online in the past year, by sex (2010–2012)

	Men reporting one or more new sexual partner, past year (n=1702)			Women reporting one or more new sexual partner, past year (n=1776)		
	Did not use the internet to find sex partners	Reported using the internet to find sexual partners	p Value	Did not use the internet to find sex partners	Reported using the internet to find sexual partners	p Value
*Health seeking behaviour*						
Attended sexual health clinic, past year						
Reported prevalence % (95% CI)	36.3 (31.5 to 41.4)	42.9 (33.7 to 52.5)		46.5 (42.2 to 50.9)	46.2 (32.9 to 60.0)	
OR (95% CI)	1.00	1.32 (0.84 to 2.07)	0.2305	1.00	0.99 (0.55 to 1.77)	0.9665
Age-adjusted OR (95% CI)	–	2.21 (1.30 to 3.77)	0.0036	–	1.71 (0.86 to 3.40)	0.1272
aOR* (95% CI)	–	1.57 (0.91 to 2.7)†	0.1070	–	1.51 (0.75 to 3.05)	0.25
Denominators‡	454, 369	143, 120		730, 412	85, 56	
HIV test, past year						
Reported prevalence % (95% CI)	7.6 (6.1 to 9.5)	19.0 (14.3 to 24.9)		13.4 (11.5 to 15.5)	17.3 (11.2 to 25.7)	
OR (95% CI)	1.00	2.86 (1.87 to 4.37)	<0.0001	1.00	1.35 (0.79 to 2.31)	0.2703
Age-adjusted OR (95% CI)	–	3.07 (1.96 to 4.78)	<0.0001	–	1.77 (1.03 to 3.06)	0.0400
aOR* (95% CI)	–	2.24 (1.39 to 3.60)†	0.0010	–	1.44 (0.81 to 2.55)	0.2106
Denominators‡	1346, 1161	274, 250		1494, 924	160, 104	
Chlamydia test, past year (aged 16–44 years)						
Reported prevalence % (95% CI)	31.4 (28.5 to 34.5)	37.0 (29.9 to 44.8)		50.3 (47.1 to 53.5)	49.4 (38.8 to 60.0)	
OR (95% CI)	1.00	1.28 (0.91 to 1.81)	0.1558	1.00	0.96 (0.62 to 1.50)	0.8690
Age-adjusted OR (95% CI)	–	1.88 (1.30 to 2.73)	0.0009	–	1.5 (0.92 to 2.45)	0.1037
aOR* (95% CI)	–	1.37 (0.93 to 2.03)†	0.1116	–	1.16 (0.69 to 1.95)	0.5763
Denominators‡	1203, 943	233, 196		1417, 809	139, 83	
Sexual health outcomes						
STI diagnosis, past year						
Reported prevalence % (95% CI)	2.0 (1.3 to 3.0)	5.7 (3.6 to 9.0)		0.042 (3.1 to 5.7)	0.016 (0.5 to 4.5)	
OR (95% CI)	1.00	2.93 (1.54 to 5.58)	0.0011	1.00	0.36 (0.12 to 1.11)	0.0751
Age-adjusted OR (95% CI)	–	3.49 (1.71 to 7.11)	0.0006	–	0.56 (0.18 to 1.78)	0.3282
aOR* (95% CI)	–	2.36 (1.02 to 5.45)†	0.0446	–	0.4 (0.12 to 1.31)	0.1302
Denominators‡	1401, 1200	279, 255		1587, 977	168, 110	
Emergency contraception use with a partner, last year						
Reported prevalence % (95% CI)	6.4 (5.2 to 7.8)	8.6 (5.7 to 12.7)		0.071 (5.6 to 8.9)	0.105 (5.8 to 18.2)	
OR (95% CI)	1.00	1.37 (0.86 to 2.20)	0.1892	1.00	1.53 (0.79 to 2.98)	0.2067
Age-adjusted OR (95% CI)	–	1.95 (1.18 to 3.22)	0.0095	–	2.25 (1.13 to 4.49)	0.0212
aOR* (95% CI)	–	1.45 (0.88 to 2.39)	0.1468	–	1.53 (0.78 to 3.00)	0.2197
Denominators‡	1384, 1190	259, 234		1565, 965	167, 109	

*Adjusted for age, partner number.

†Additionally adjusted for reporting a same-sex partner in the past year.

‡Unweighted, weighted.

In the wider sexually experienced population, stronger associations between sexual health outcomes and reporting finding partners online were observed for men, and associations with several health seeking behaviours and poor sexual health outcomes were also observed for women (see online supplement 2).

### Urine-based STI testing

STI testing data were available for 815 men and 853 women aged 16–44 years who reported a new partner in the past year. Among these participants, 12 out of 139 men (9.3% (4.5 to 18.3)) and 7 out of 82 (6.2% (2.8 to 12.0)) women who reported finding partners online had a non-viral STI detected (*Chlamydia trachomatis*, *Neisseria gonorrhoeae*, *Mycoplasma genitalium*, *Trichomonas vaginalis*), compared with 23 out of 676 men (3.2% (2.0 to 5.2) and 56 out of 771 (5.7% (4.3 to 7.5)) women who did not use the internet to find a partner. After adjusting for age, there was evidence of a weak association for men reporting use of the internet to find partners and detection of a non-viral STI (aOR 2.59 (1.0 to 6.8)), but there was no association for women (aOR 1.37 (0.6 to 3.3)).

## Discussion

### Principal findings

Around 1 in 6 men and 1 in 10 women with at least one new sexual partner in the past year reported using the internet to find sexual partners, and this was most commonly reported among adults aged 35–44 years. Reporting a non-heterosexual identity was strongly associated with finding partners online, as were reporting sexual risk behaviours for STIs and having a higher perceived risk of HIV and STIs. However, after adjusting for key behavioural confounders, we found weaker associations in men and no associations in women between using the internet to find sexual partners and sexual health clinic attendance or STI testing. These data suggest a mismatch between need for (table 3) and uptake (table 4) of sexual health services in those using the internet to find sexual partners, who might be at higher risk of STIs.

After adjusting for age, we observed a positive association between using the internet to find sexual partners and detection of a non-viral STI for men, but not for women, however the data should be interpreted with caution given the small numbers.

### Strengths and limitations

To our knowledge, we present the first population-based study in a broad age-range to examine associations between finding partners online and sociodemographic factors, markers of sexual risk and sexual health outcomes. These data are from a national probability survey, which avoids the selection bias of convenience and clinic samples.

This study has several limitations. The data for Natsal-3 were collected between 2010 and 2012, and in the intervening time, new platforms for finding partners online have emerged and been adopted, and our observations might not reflect the current situation in this fast changing field. While the Natsal-3 fieldwork was being undertaken, dating-apps focusing on MSM, such as Grindr and Scruff were available, while dating-apps targeted at wider populations emerged later.

The location-based dating-app Tinder was launched in September 2012, after Natsal-3 fieldwork was complete, and by 2014 had amassed an estimated 50 million (mostly heterosexual) users worldwide.^21^ The subsequent increase of available apps has likely changed the way people use the internet to find partners. While our study will not have captured the social and behavioural changes that have occurred as new technologies have emerged, by examining the use of the internet to find partners at a point where the behaviour was relatively rare in the population, we have highlighted the need for further research in this field, and provided data for describing trends in future research conducted on a population-level.

Although Natsal-3 collected data on a wide range of sexual behaviours and health outcomes, questions concerning use of the internet to find sexual partners were limited. We do not know whether participants who reported using the internet to find sexual partners actually had sex with a partner met online. We also do not have event-level information on whether participants exhibited different behaviours with online versus offline partners, or whether different sexual health outcomes resulted from encounters with online partners. Comparisons of individuals' encounters with their offline and online partners might help to determine whether observed risk behaviours are associated with the individual, or with the internet itself.^22^ The study was not designed to determine whether the internet is a marker for risk in general, or whether finding partners online is inherently risky, and due to the cross-sectional nature of the data, neither causality nor directionality can be inferred.

### Comparison with other studies

The finding that more men than women use the internet to find sexual partners is consistent with clinic studies^7^ ^9^ and estimates from the second Australian Study of Health and Relationships (ASHR2), also a national probability sample survey, undertaken in men and women aged 16–69 years in 2012–2013.^23^ Prevalence estimates for use of the internet to look for potential partners in the past year were higher in ASHR2 than in the comparable Natsal-3 population (7.0% of men and 3.8% of women with a sexual partner in the past year, aged 16–69 years vs 5.6% of men and 2.5% of women with a sexual partner in the past year, aged 16–69 years); however, these results are not directly comparable. In ASHR2, participants were asked specifically about both website and app use to find potential partners ever, and in the past year. Additionally, data collection took place a year later than for Natsal-3, and so this higher estimate of prevalence might be due to the increased availability of dating-apps. Unlike our study, ASHR2 asked whether participants had sex with a partner met online, and these estimates were lower than those of participants looking for partners online (2.5% of men and 1.3% of women). However, our study reported associations between finding partners online and sociodemographic factors, markers of sexual risk and sexual health outcomes, whereas ASHR2 did not.

Use of the internet to find sexual partners has been well described for MSM, and our findings were consistent with other reports;^7^ ^9^ we observed that MSM were more likely to use the internet to find partners than heterosexual men and women. Studies including participants with diverse sexual orientations have stratified the analysis to account for baseline differences in risk behaviour between different groups. However, in our population-based data non-heterosexual groups were too small to do this. Nevertheless, we were able to adjust for reporting a same-sex partner for men when investigating associations for risk perception and sexual health outcomes.

We observed associations between sexual risk behaviour and using the internet to find partners, which has previously been reported in convenience samples of MSM,^8^ ^24^ ^25^ young heterosexuals^11^ ^w6^ and adult populations.^9^ In many of these studies, the risks were observed to be independent of meeting venue, and attributable to the behaviour of the individual. For example, Bolding *et al*^9^ found that individuals exhibited high-risk sexual behaviour both with partners met online and offline. Similarly, in a study of MSM, associations between high-risk sexual behaviour and internet partners was attributed to multiple partnerships and more commonly identified in individuals with both online and offline partners when compared with individuals with exclusively online or offline partners, although the observed differences were small.^25^ Gravningen *et al*.^11^ observed that sexual risk behaviours among youths were associated with reporting using the internet to find a sex partner, as opposed to seeking a partner for a romantic relationship. In turn, each of these studies have suggested that associations between high-risk sexual behaviour and internet partners are more likely to be due to the individual's risk behaviour in general, rather than the internet being a risk environment, per se.

### Meaning of the study and implications

This study has identified an important group within the population who exhibit higher sexual behavioural risk and risk perception, although it is unclear whether internet-use is the cause or a marker for increased sexual risk. There is evidence that the internet facilitates disassortative mixing among MSM,^3^ ^8^ and it might be that the same is true among heterosexuals and women who have sex with women such that the population using the internet to find partners might be a bridging population for the transmission of STIs between higher and lower risk groups. This population might also be targeted for health promotion campaigns and interventions through the same medium that is being used to access partners. Sexual health information and advice is increasingly available online,^26^ ^27^ and although our study preceded the widespread use of dating-apps, other studies have explored their potential to be used for STI prevention. For example, among MSM, dating-apps have been shown to enhance partner notification,^28^ and it has been suggested that integrating HIV prevention interventions into dating-apps might allow for targeting of individuals who exhibit markers of risk in their individual profiles.^29^

Awareness campaigns might be of particular importance to older adults who have recently acquired a new sexual partner, because this study has highlighted the internet as an important source of new partners for adults aged 35–44 years. In our study population, approximately one in three men and one in six women aged 35–44 years reported using the internet to find a sexual partner. Older adults (aged 35–44 years) are less likely to attend sexual health clinics^16^ and might have received inadequate sexual health education as young adults.^30^

### Unanswered questions and future research

Further studies in the general population are needed to determine whether finding partners online is a risk in itself, or if it is a strategy more commonly adopted by individuals who exhibit riskier sexual behaviour. In survey research, this might be achieved by including more detailed questions, enabling event-level analyses about sexual behaviours with, and characteristics of, online and offline partners. Questions concerning the specific internet meeting site or app used to find a sexual partner will be important to assess whether risks are associated with the internet generally or with specific online platforms for meeting partners, which might help target intervention strategies.

## Conclusion

Using the internet to find sexual partners was more common among men than women in 2010–2012, and was strongly associated with reporting sexual risk, which might be important for clinical risk assessment. Since then, the range and availability of opportunities for finding sexual partners online have increased considerably and so these data might underestimate the public health significance of this phenomenon. Further in-depth research is needed to understand the extent of the risks associated with finding partners online, whether these risks are at the individual or partnership level, and the potential for tailored interventions to inform STI control.

**Key messages**

- This study investigated the prevalence of, and factors associated with, use of the internet to find sexual partners using data from a national probability sample survey.
- Finding sexual partners online was reported by around 1 in 6 men and 1 in 10 women (aged 16–74 years) with one or more new sexual partner(s) in the past year.
- It was associated with reporting sexual risk behaviour in men and women, while associations with sexual health service use outcomes were observed for men only.
- These data suggest a mismatch between need for and uptake of sexual health services in those using the internet to find sexual partners, who might be at higher risk of STIs.
- There has been a considerable increase in online venues to meet sexual partners, and these data are therefore important for describing trends in future research.
