# Supplementary material for: Finding sexual partners online: prevalence and associations with sexual behaviour, STI diagnoses and other sexual health outcomes in the British population
Source: Sex Transm Infect. 2017 Apr 10;93(8):572–82. doi: 10.1136/sextrans-2016-052994 (PMC5739860; doi:10.1136/sextrans-2016-052994)
Supplement: supplement 1 [file sextrans-2016-052994supp001.pdf]

Online supplement 1. Reporting finding partners online in the past year in relation to sexual behaviour and risk perception, for men and women reporting one or more sexual partners, ever

	Men reporting one or more sexual partners, ever (n=5698)				Women reporting one or more sexual partners, ever (n=8198)			
	**Prevalence %(95%CI*)**	**Crude OR[1] (95% CI[4])**	**Adjusted OR[1,2] (95%CI[4])**	**Denominator[5]**	**Prevalence %(95%C[4])**	**Crude OR[1] (95% CI[4])**	**Adjusted OR[1,2] (95%CI[4])**	**Denominator[5]**
**Sexual risk behaviour**								
**No. of sexual partners, past year**		p<0.0001	p<0.0001			p<0.0001	p<0.0001	
1	1.8 (1.5-2.3)	0.45 (0.29-0.71)	0.42 (0.26-0.67)	3596, 4855	1.1 (0.9-1.4)	0.67 (0.42-1.09)	0.73 (0.40-1.33)	5470, 5041
2	11.3 (8.6-14.8)	3.1 (1.90-5.07)	2.73 (1.57-4.76)	543, 517	6.7 (4.5-9.9)	4.16 (2.34-7.37)	4.75 (2.18-10.38)	574, 366
3-4	21.6 (17.2-26.7)	6.68 (4.09-10.90)	5.83 (3.31-10.27)	414, 364	14.2 (10.5-19.0)	9.64 (5.65-16.46)	11.25 (5.06-24.99)	413, 230
5+	34.5 (28.3-41.3)	12.78 (7.88-20.75)	11.19 (6.36-19.67)	306, 265	20.3 (14.2-28.1)	14.77 (8.17-26.69)	17.44 (7.32-41.52)	229, 137
**Same sex partner, past year**		p<0.0001	p<0.0001			p<0.0001	p<0.0001	
No	4.3 (3.8-4.9)	1.00	1.00	5537, 6795	2.2 (1.9-2.5)	1.00	1.00	7980, 6999
Yes	41.9 (33.2-51.2)	15.94 (10.72-23.71)	15.58 (10.36-23.44)	161, 161	11.7 (7.3-18.2)	5.98 (3.50-10.20)	5.06 (2.93-8.73)	216, 160
**Condomless sex with ≥2 partners, past year**		p<0.0001	p<0.0001			p<0.0001	p<0.0001	
No	4.22 (3.7-4.8)	1.00	1.00	5260, 6474	1.9 (1.6-2.2)	1.00	1.00	7634, 6776
Yes	19.2 (14.9-24.4)	5.4 (3.87-7.53)	5.24 (3.71-7.39)	382, 408	12.6 (9.4-16.9)	7.58 (5.23-10.98)	6.36 (4.25-9.53)	483, 299
**Condomless sex on 1st occasion with most recent partner**		p=0.9820	p=0.0956			p=0.6318	p=0.5565	
No	4.1 (3.4-4.9)	1.00	1.00	2441, 3311	2.0 (1.6-2.5)	1.00	1.00	3394, 3206
Yes	4.1 (3.4-5.0)	1 (0.75-1.34)	1.29 (0.96-1.74)	2317, 2710	1.8 (1.4-2.4)	0.92 (0.66-1.29)	1.11 (0.78-1.60)	3741, 3103
**Concurrent partners, past year**		p<0.0001	p<0.0001			p<0.0001	p<0.0001	
No	3.2 (2.7-3.8)	1.00	1.00	4005, 5194	1.7 (1.4-2.1)	1.00	1.00	5841, 5255
Yes	23.9 (19.4-28.9)	9.51 (6.93-13.05)	8.63 (6.18-12.06)	460, 452	13.6 (9.9-18.5)	9.01 (5.99-13.54)	8.84 (5.63-13.89)	417, 249
≥2 partners, unknown if overlapping	12.5 (9.4-16.5)	4.34 (3.01-6.26)	3.9 (2.65-5.73)	436, 406	6.9 (4.4-10.6)	4.22 (2.55-7.01)	4.17 (2.46-7.07)	477, 315

		p<0.0001	p<0.0001			p=0.0013	p=0.0059	
**Taken drugs to assist sexual performance**	4.2 (3.7-4.7)	1.00	1.00	4890, 5972	2.3 (2.0-2.7)	1.00	1.00	7894, 6926
No	12.5 (10.1-15.4)	3.29 (2.49-4.35)	4.05 (3.05-5.38)	723, 883	5.9 (3.4-9.9)	2.61 (1.46-4.69)	2.27 (1.27-4.07)	198, 138
Yes		p<0.0001	p<0.0001			-	-	
**Paid money for sex, past year[6]**	4.9 (4.4-5.5)	1.00	1.00	5608, 6855	-	-	-	-
No	31.3 (20.2-45.3)	8.87 (4.80-16.39)	9.4 (5.02-17.59)	71, 81	-	-	-	-
Yes								
**Risk perception**		p<0.001	p<0.0001			p<0.0001	p<0.0001	
**HIV/AIDS risk: to self**	2.9 (2.4-3.4)	1.00	1.00	4013, 5242	1.4 (1.2-1.7)	1.00	1.00	6499, 5906
Not at all at risk	11.8 (10.0-13.8)	4.52 (3.50-5.84)	3.15 (2.38-4.18)[3]	1480, 1519	6.8 (5.5-8.4)	5.12 (3.79-6.92)	4.62 (3.37-6.34)	1495, 1092
Not very much	18.6 (13.1-25.8)	7.75 (4.97-12.08)	4.7 (2.99-7.39)[3]	169, 153	9.8 (5.4-17.2)	7.6 (3.92-14.72)	6.96 (3.61-13.45)	158, 124
Greatly/quite a lot at risk		p<0.001	p<0.0001			p<0.0001	p<0.0001	
**Other STI risk: to self**	2.5 (2.1-3.0)	1.00	1.00	3821, 5084	1.4 (1.1-1.7)	1.00	1.00	6276, 5794
Not at all at risk	10.4 (8.9-12.2)	4.57 (3.49-5.99)	3.4 (2.53-4.58)[3]	1544, 1567	6.1 (4.8-7.6)	4.67 (3.42-6.36)	4.3 (3.09-5.99)	1624, 1149
Not very much	24.5 (19.0-30.9)	12.72 (8.71-18.57)	8.31 (5.54-12.46)[3]	300, 266	11.4 (8.0-16.1)	9.33 (6.00-14.49)	8.52 (5.43-13.37)	256, 180
Greatly/quite a lot at risk		p<0.0001	p<0.0001			p<0.0001	p<0.0001	

Notes for Online Supplement 1

[1] OR= Odds Ratio

[2] Adjusted for Age

[3] Adjusted for reporting a same sex partner in the past year

[4] CI= Confidence Interval

[5] Unweighted, weighted

[6] Outcome reported for Men only

Online supplement 2. Reporting of sexual health outcomes and health seeking behaviours in relation to finding partners online in the past year, for men and women reporting one or more sexual partners, ever

	Men reporting one or more sexual partners, ever (n=5698)			Women reporting one or more sexual partners, ever (n=8198)		
	**Did not use the internet to find sex partners**	**Reported using the internet to find sexual partners**	**p-value**	**Did not use the internet to find sex partners**	**Reported using the internet to find sexual partners**	**p-value**
**Health Seeking Behaviour**						
**Attended sexual health clinic, past year**						
Reported Prevalence % (95% CI[1])	17.8 (15.8-20.1)	39 (31.3-47.2)		21.2 (19.30-23.10)	41 (29.4-53.8)	
OR[2] (95% CI[1])	1.00	2.94 (2.03-4.27)	p<0.0001	1.00	2.59 (1.53-4.38)	0.0004
Age-Adjusted OR (95% CI[1])	-	3.48 (2.27-5.33)	p<0.0001	-	4.14 (2.06-8.33)	0.0001
AOR[2,3] (95%CI[1])	-	1.66 (1.03-2.68)[4]	0.0378	-	2.25 (1.08-4.69)	0.0312
Denominators[5]	1233, 1312	177, 150		2198, 1522	107, 74	
**HIV test, past year**						
Reported Prevalence % (95% CI[1])	3.0 (2.5-3.6)	15.0		5.3 (4.8-5.8)	13.7 (9.2-19.8)	
OR[2] (95% CI[1])	1.00	5.7 (3.98-8.18)	p<0.0001	1.00	2.83 (1.79-4.47)	p<0.0001
Age-Adjusted OR (95% CI[1])	-	4.58 (3.17-6.61)	p<0.0001	-	2.27 (1.38-3.76)	0.0014
AOR[2,3] (95%CI[1])	-	2.26 (1.44-3.57)[4]	0.0005	-	1.82 (1.09-3.03)	0.0229
Denominators[5]	5058, 6303	372, 341		7492, 6601	234, 160	
**Chlamydia test, past year (16-44 year olds)**						
Reported Prevalence % (95% CI[1])	14.8 (13.6-16.0)	32.3% (26.6-38.7)		25.3 (24.0-26.7)	47% (38.2-55.9)	
OR[2] (95% CI[1])	1.00	2.75 (2.06-3.67)	p<0.0001	1.00	2.61 (1.82-3.75)	p<0.0001
Age-Adjusted OR (95% CI[1])	-	2.84 (2.03-3.97)	p<0.0001	-	3.19 (1.98-5.13)	p<0.0001
AOR[2,3] (95%CI[1])	-	1.62 (1.12-2.32)[4]	p=0.0095	-	1.88 (1.14-3.10)	0.0139
Denominators[5]	3295, 3417	306, 257		5092, 3562	188, 116	
**Sexual Health Outcomes**						
**STI[6] diagnosis, past year**						
Reported Prevalence % (95% CI[1])	0.8 (0.6-1.0)	4.1 (2.6-6.6)		0.9 (0.7-1.1)	1.6 (0.6-4.2)	
OR[2] (95% CI[1])	1.00	5.6 (3.10-10.11)	p<0.0001	1.00	1.82 (0.65-5.07)	0.253

Age-Adjusted OR (95% CI[1])	-	4.16 (2.30-7.51)	p<0.0001	-	1.44 (0.51-4.10)	0.4948
AOR[2,3] (95%CI[1])	-	1.68 (0.71-3.97)[4]	0.234	-	0.68 (0.22-2.12)	0.5091
Denominators[5]	5208, 6461	378, 351		7824, 6870	244, 167	
**Emergency contraception use with a partner, last year**						
Reported Prevalence % (95% CI[1])	2.1 (1.8-2.5)	6.7 (4.5-9.7)		1.6 (1.4-1.9)	7.5 (4.3-12.8)	
OR[2] (95% CI[1])	1.00	3.34 (2.16-5.14)	p<0.0001	1.00	4.94 (2.75-8.87)	p<0.0001
Age-Adjusted OR (95% CI[1])	-	2.51 (1.63-3.86)	p<0.0001	-	4.2 (2.22-7.98)	p<0.0001
AOR[2,3] (95%CI[1])	-	1.44 (0.92-2.25)	0.1119	-	2.14 (1.13-4.05)	0.0194
Denominators[5]	5175, 6451	356, 329		7795, 6870	243, 167	

Notes for Online Supplement 2

[1]CI= Confidence Interval

[2] OR= Odds Ratio

[3] Adjusted for age, partner number

[4] Additionally adjusted for reporting a same-sex partner in the past year

[5] Unweighted, weighted

[6] STI = Sexually Transmitted Infection
