# Supplementary material for: Finding sexual partners online: prevalence and associations with sexual behaviour, STI diagnoses and other sexual health outcomes in the British population
Source: Sex Transm Infect. 2017 Apr 10;93(8):572–82. doi: 10.1136/sextrans-2016-052994 (PMC5739860; doi:10.1136/sextrans-2016-052994)
Supplement: Supplementary appendix [file sextrans-2016-052994supp002.pdf]

Appendix 2: Web References

w1. Bull SS, McFarlane M. Soliciting sex on the Internet: what are the risks for sexually transmitted diseases and HIV? *Sex Transm Dis* 2000;**27**:545–50.

w2. Lehmiller JJ, Ioerger M. Social networking smartphone applications and sexual health outcomes among men who have sex with men. *PLoS One* 2014;**9**:e86603. doi:10.1371/journal.pone.0086603

w3. Elford J, Bolding G, Davis M, *et al.* Web-based behavioral surveillance among men who have sex with men: a comparison of online and offline samples in London, UK. *J Acquir Immune Defic Syndr* 2004;**35**:421–6.

w4. Hull P, Mao L, Prestage G, *et al.* The use of mobile phone apps by Australian gay and bisexual men to meet sex partners: an analysis of sex-seeking repertoires and risks for HIV and STIs using behavioural surveillance data. *Sex Transm Infect* 2016;:sextrans-2015-052325. doi:10.1136/sextrans-2015-052325

w5. Bolding G, Davis M, Hart G, *et al.* Gay men who look for sex on the Internet: is there more HIV/STI risk with online partners? *AIDS* 2005;**19**:961–8.

w6 . Buhi ER, Klinkenberger N, McFarlane M, *et al.* Evaluating the Internet as a sexually transmitted disease risk environment for teens: findings from the communication, health, and teens study. *Sex Transm Dis* 2013;**40**:528–33. doi:10.1097/OLQ.0b013e31829413f7

w7. Ybarra ML, Mitchell KJ. A National Study of Lesbian, Gay, Bisexual (LGB), and Non-LGB Youth Sexual Behavior Online and In-Person. *Arch Sex Behav* Published Online First: 18 April 2015. doi:10.1007/s10508-015-0491-7

w8. Field N, Clifton S, Alexander S, *et al.* Confirmatory assays are essential when using molecular testing for *Neisseria gonorrhoeae* in low-prevalence settings: insights from the third National Survey of Sexual Attitudes and Lifestyles (Natsal-3). *Sex Transm Infect* 2014.

w9. Sonnenberg P, Ison CA, Clifton S, *et al.* Epidemiology of *Mycoplasma genitalium* in British men and women aged 16–44 years: evidence from the third National Survey of Sexual Attitudes and Lifestyles (Natsal-3). *Int J Epidemiol* 2015;**44**:1982–94. doi:10.1093/ije/dyv194

w10. Field N, Clifton S, Alexander S, *et al.* *Trichomonas vaginalis* infection is uncommon in the British general population: implications for clinical testing and public health screening. *Sex Transm Infect* 2016.
